# Procalcitonin in early allograft dysfunction after orthotopic liver transplantation: a retrospective single centre study

**DOI:** 10.1186/s12876-022-02486-5

**Published:** 2022-08-31

**Authors:** Katja Frick, Elisabeth A. Beller, Marit Kalisvaart, Philipp Dutkowski, Reto A. Schüpbach, Stephanie Klinzing

**Affiliations:** 1 Institute of Intensive Care Medicine, University Hospital of Zurich, Raemistrasse 100, 8091 Zurich, Switzerland; 2 Department of Surgery and Transplantation, University Hospital of Zurich, Raemistrasse 100, 8091 Zurich, Switzerland

**Keywords:** Orthotopic liver transplantation, Early allograft dysfunction, Primary nonfunction, Ischemia–reperfusion injury, Procalcitonin, Outcome, Donation after brain death, Donation after cardiac death

## Abstract

**Background:**

Ischemia–reperfusion injury (IRI) is the pathophysiological hallmark of hepatic dysfunction after orthotopic liver transplantation (OLT). Related to IRI, early allograft dysfunction (EAD) after OLT affects short- and long-term outcome. During inflammatory states, the liver seems to be the main source of procalcitonin (PCT), which has been shown to increase independently of bacterial infection. This study investigates the association of PCT, IRI and EAD as well as the predictive value of PCT during the first postoperative week in terms of short- and long-term outcome after OLT.

**Methods:**

Patients ≥ 18 years undergoing OLT between January 2016 and April 2020 at the University Hospital of Zurich were eligible for this retrospective study. Patients with incomplete PCT data on postoperative days (POD) 1 + 2 or combined liver-kidney transplantation were excluded. The PCT course during the first postoperative week, its association with EAD, defined by the criteria of Olthoff, and IRI, defined as aminotransferase level > 2000 IU/L within 2 PODs, were analysed. Finally, 90-day as well as 12-month graft and patient survival were assessed.

**Results:**

Of 234 patients undergoing OLT, 110 patients were included. Overall, EAD and IRI patients had significantly higher median PCT values on POD 2 [31.3 (9.7–53.8) mcg/l vs. 11.1 (5.3–25.0) mcg/l; *p* < 0.001 and 27.7 (9.7–51.9) mcg/l vs. 11.5 (5.5–25.2) mcg/l; *p* < 0.001] and impaired 90-day graft survival (79.2% vs. 95.2%; *p* = 0.01 and 80.4% vs. 93.8%; *p* = 0.033). IRI patients with PCT < 15 mcg/l on POD 2 had reduced 90-day graft and patient survival (57.9% vs. 93.8%; *p* = 0.001 and 68.4% vs. 93.8%; *p* = 0.008) as well as impaired 12-month graft and patient survival (57.9% vs. 96.3%; *p* = 0.001 and 68.4% vs. 96.3%; *p* = 0.008), while the outcome of IRI patients with PCT > 15 mcg/l on POD 2 was comparable to that of patients without IRI/EAD.

**Conclusion:**

Generally, PCT is increased in the early postoperative phase after OLT. Patients with EAD and IRI have a significantly increased PCT maximum on POD 2, and impaired 90-day graft survival. PCT measurement may have potential as an additional outcome predictor in the early phase after OLT, as in our subanalysis of IRI patients, PCT values < 15 mcg/l were associated with impaired outcome.

**Supplementary Information:**

The online version contains supplementary material available at 10.1186/s12876-022-02486-5.

## Background

Early allograft dysfunction (EAD) is a frequent complication during the first postoperative week after orthotopic liver transplantation (OLT), occurring in up to 36% of patients [1]. EAD has the potential for full graft recovery, but deteriorates to primary nonfunction (PNF) in up to 7% of cases, where hepatic failure during the early post-transplant course will lead to death without immediate retransplantation [1–3]. Nevertheless, EAD significantly affects short- and long-term morbidity and mortality after OLT [4, 5]. EAD is considered a consequence of ischemia–reperfusion injury (IRI). Donor associated risk factors for EAD are extended donor criteria like age > 70 years, hepatic steatosis > 30%, BMI > 30 kg/m^2^, hypotensive episodes with high vasopressor requirement and donor cardiac arrest or donation after cardiac death (DCD) [2, 6]. Furthermore, prolonged ischemic times as well as recipients’ preoperative illness severity with necessary organ support are associated factors [2, 7]. While PNF is commonly defined as hepatic failure leading to graft loss within the first postoperative week, to date no uniform definition of EAD has been accepted. Nevertheless, there is general agreement that aminotransferase levels reflect graft injury, while bilirubin and international normalized ratio (INR) predict metabolic graft function [8]. A widely used definition by Olthoff proposes EAD defined by the laboratory values of serum bilirubin, transaminases and INR during the first 7 days after OLT [5].

Procalcitonin (PCT) is the 116 amino acid precursor peptide of calcitonin. In health, it is produced by thyroidal C-cells encoded by the CALC-1 gene on chromosome 11 and is detectable in serum usually at concentrations below 0.1 mcg/l [9]. PCT is an established biomarker of severe bacterial infection, reaching its highest values during septic shock. It is upregulated in several body tissues during inflammation [10], but the liver seems to be the major source of PCT [11–15]. This was concluded in an endotoxin shock model, where PCT increase was significantly lower in hepatectomized animals [13].

Increased PCT levels are observed independent of bacterial infection, e.g. in the setting of major abdominal surgery [16]. PCT during OLT has been investigated in several studies. In uncomplicated courses, PCT seems to peak on the first or second postoperative day (POD), afterwards normalizing within the first postoperative week [17–21]. Whether this is due to the general inflammatory response [16] or additionally affected by an inflammatory cascade related to IRI [7, 22] remains to be clarified.

Studies investigating the diagnostic ability of PCT to predict EAD have yielded contradictory results. While no difference in peak PCT was found in recipients with and without EAD in one study [20], significantly higher PCT levels in a paediatric liver transplant population developing EAD were described elsewhere [23].

As data concerning the association of PCT, EAD and IRI are sparse; the aim of our study is to gain further knowledge concerning this association and to investigate the possible predictive value of PCT in terms of morbidity and mortality. We hypothesize that the procedure-related non-infectious inflammation generated in the allograft itself affects PCT serum level in the early post-transplant period and, with that, short- and long-term outcome after liver transplantation.

## Methods

### Study design

Patients ≥ 18 years undergoing deceased-donor liver transplantation from January 2016 to April 2020 at the University Hospital of Zurich were eligible for this retrospective study. Patients undergoing combined liver-kidney transplantation and patients with incomplete data of PCT on POD 1 and 2 were excluded. The study was approved by the Institutional Ethics Committee of Zurich (BASEC-Nr. 2020-00188). All patients gave written informed consent for data analysis before transplantation.

### Operative and immunosuppressive management

Donation after brain death (DBD) livers were procured by the standard retrieval protocol. DCD livers were harvested by the super rapid retrieval technique, followed by cold storage with Institute-George-Lopez-1 solution. DCD and marginal DBD liver grafts underwent hypothermic oxygenated machine perfusion (HOPE) treatment during recipient hepatectomy according to institutional practice [24]. Organ implantation was performed according to the centre routine approach using classic cava-replacement technique without veno-venous bypass. Reperfusion was initiated through the portal vein with subsequent arterial reperfusion. Immunosuppression was applied corresponding to centre guidelines with methylprednisolone switched to prednisolone on POD 6 and tacrolimus started between POD 1–5 adjusted to kidney function. Patients with a glomerular filtration rate (GFR) < 40 ml/min as well as DCD recipients received basiliximab induction and repetition on POD 4.

### PCT measurement

PCT was measured in the daily morning routine laboratory during the first posttransplant week after OLT. Duration of measurement was dependent on the clinical course of the patient. In uncomplicated courses PCT was measured predominantly on POD 1–3. During the first year of the study period PCT was mainly measured in complicated cases. These are the reasons, why we choose to only include patients with a complete dataset of PCT on POD 1 + 2 into the study. Preoperatively PCT was not routinely measured at our institution.

### Data collection and outcome parameters

Electronic patient records were screened and baseline demographic patient characteristics as well as graft specific and operative data were noted. Preoperative and daily laboratory data during the first postoperative week as well as data on the early postoperative course until hospital discharge were also collected. Laboratory data included C-reactive protein (CRP), white blood cell (WBC) count, PCT, ALT, AST, INR, creatinine and GFR. Data on the early postoperative course included overall major complications ≥ stage 3B according to the Clavien-Dindo-Classification [25], postoperative infections, immunosuppressive agents, acute rejection episodes, the occurrence of acute kidney failure plus the need for renal replacement therapy (RRT), arterial and biliary complications, the need for relaparotomy and the length of intensive care unit (ICU)- and hospital stay.

The follow up period was 12 months. The primary outcome for analysis was EAD, defined by the criteria of Olthoff: ALT or AST > 2000 IU/L within 7 PODs, bilirubin ≥ 10 mg/dL and/or INR ≥ 1.6 on POD 7. PNF was defined by death or the need for retransplantation within 7 days after the initial procedure. Secondary outcome was IRI, defined as AST/ALT > 2000 IU/L within POD 1 or 2. Furthermore, 90-day as well as 12-month graft and patient survival were assessed.

### Statistical analysis

Data were analysed with IBM SPSS Statistics version 26 (IBM Corporation, Armonk, NY). Continuous variables were analysed using the Mann–Whitney U test. To compare categorical variables, the Chi-square test or the Fisher exact test were used. P values less than 0.05 were considered statistically significant. Continuous variables were expressed as median with interquartile range. Categorical variables were expressed in quantities and percentages. For analysis of short- and long-term outcome according to PCT, patients with signs of IRI on the first 2 PODs were divided into 2 groups using the median PCT level. Long-term survival rates were estimated using Kaplan–Meier methods, with comparisons between groups performed using Log-rank tests.

## Results

**Fig. 1:**
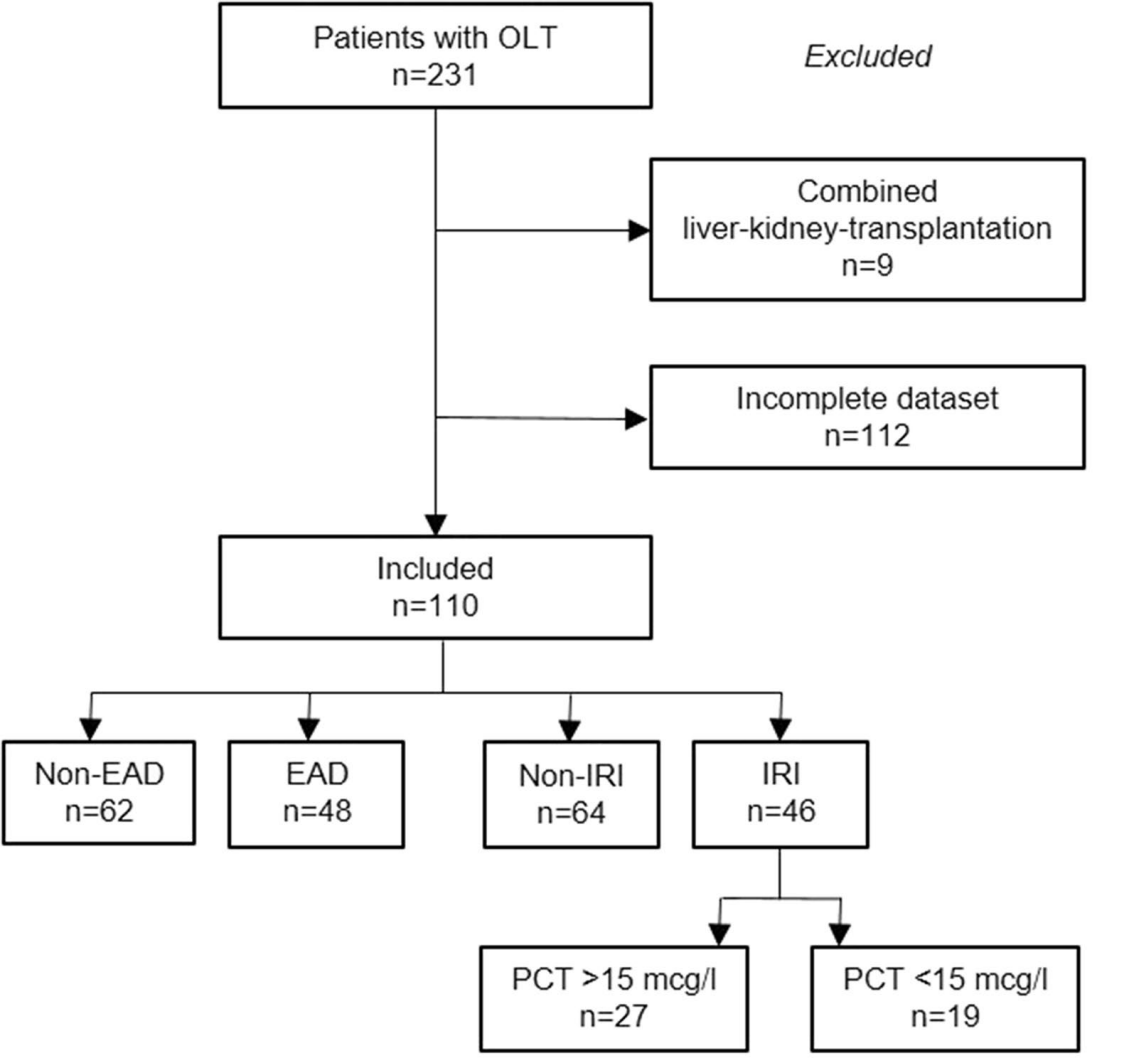
Patient flow diagram. A total of 231 patients were eligible for study purposes. After exclusions, data from 110 patients were included. Early allograft dysfunction (EAD) was defined according to the Olthoff criteria. Elevated aminotransferase level on the first 2 postoperative days (AST or ALT > 2000 IU/L) was defined as a sign of ischemia–reperfusion-injury (IRI). IRI patients were classified according to their PCT level into IRI patients with PCT > 15 mcg/l and IRI patients with PCT < 15 mcg/l

From January 1st 2016 to April 30th 2020, 231 patients underwent 234 deceased-donor liver transplantations at the University Hospital of Zurich. Of these, 110 patients with a complete dataset of PCT on POD 1 + 2 were included in the study. In all, 121 patients were excluded due either to missing PCT values on POD 1 + 2 (n = 112) or to combined liver-kidney transplantation (n = 9). A patient flow diagram through this study is shown in Fig. 1.

Of the 110 patients with complete PCT data on POD 1 + 2 included to this study, 62 patients (56.4%) had normal graft function during the 1st week (non-EAD group), and 48 patients (43.6%) fulfilled the Olthoff criteria for EAD (EAD group), of which 4 patients developed PNF, corresponding to an incidence of 3.6% in the study cohort. Among the EAD patients, 42 (87.5%) met one criterion of Olthoff, 5 patients (10.4%) met two criteria and 1 patient (2.1%) met all three criteria. 46 of the 48 EAD patients (95.8%) showed an aminotransferase level > 2000 IU/L on POD 1 + 2, indicating IRI-group, and 9 showed a functional deficit on POD 7. Detailed data are shown in Table 1.

**Table 1:** Olthoff criteria of the study group

	AST/ALT > 2000 IU/L* (n = 46)	Bilirubin ≥ 10 mg/dL (n = 7)	INR ≥ 1.6 (n = 2)
*Number of criteria*			
1 (n = 42)	40 (95.2%)	2 (4.8%)	0
2 (n = 5)	5 (100%)	4 (80%)	1 (20%)
3 (n = 1)	1 (100%)	1 (100%)	1 (100%)

*AST/ALT peak was reached within the first 2 postoperative days after liver transplantation

### Baseline characteristics, infections and immunosuppression

Baseline characteristics for the eligible cohort (n = 231) and study cohort (n = 110) are shown in Table 2. In the study group, there was a statistically significant difference between patients meeting EAD criteria and those who did not in terms of allografts, cold ischemic time and the preoperative MELD score. DCD organs more often developed EAD [22/48 grafts (45.8%) vs. 8/62 grafts (12.9%); *p* < 0.001] and grafts of the EAD group were exposed for a longer cold ischemic time [7.4 (6.6–9.6) h vs. 6.5 (5.4–8.4) h; *p* = 0.013]. The preoperative MELD score was lower compared to the non-EAD group [17 (9–31) vs. 23 (16–34); *p* = 0.029].

**Table 2:** Baseline characteristics of EAD versus non-EAD patients

	Whole cohort (n = 231)	Study cohort (n = 110)	Non-EAD (n = 62)	EAD (n = 48)	*p*-value
*Donor and Graft*					
Age (years)	58 (47–71)	60 (48–73)	60 (48–71)	61 (50–74)	0.523
Female gender	98 (42.4%)	47 (42.7%)	29 (46.8%)	18 (37.5%)	0.329
BMI (n = 109)	25.5 (23.0–28.0)	26 (23–28)	25 (22.5–28)	26 (24–29)	0.473
Split liver transplantation	7 (3.0%)	3 (2.7%)	3 (4.8%)	0	0.255
DCD	68 (29.4%)	30 (27.3%)	8 (12.9%)	22 (45.8%)	< 0.001
Cold ischemia time (h) (n = 106)	7.0 (5.8–8.4)	7.1 (5.8–8.7)	6.5 (5.4–8.4)	7.4 (6.6–9.6)	0.013
Warm ischemia time (min)	34.0 (30.0–38.0)	34.5 (29.8–38.3)	34.5 (31.5–44.0)	33.5 (29.0–38.3)	0.504
*Recipient*					
Age (years)	57 (48–63)	57 (48–64)	57 (46–63)	58 (50–64)	0.708
Female gender	76 (32.9%)	36 (32.7%)	19 (30.6%)	17 (35.4%)	0.597
Body mass index	27.0 (22.7–30.1)	27.4 (23.5–30.9)	27.3 (23.2–30.6)	27.9 (23.9–31.7)	0.849
Charlson-Comorbidity-Index	5 (3–6)	5 (3–5)	5 (3–5)	5 (3–5)	0.606
Serum-Creatinine (µmol/l) (n = 81)	88 (68–133)	93 (68–145)	91 (71–156)	94 (66–122)	0.384
Preoperative RRT	53 (22.9%)	28 (25.5%)	17 (27.4%)	11 (22.9%)	0.591
Laboratory MELD-score (n = 109)	19 (11–31)	20 (12–33)	23 (16–34)	17 (9–31)	0.029
Liver cirrhosis (Child–Pugh-Score)					0.147
No cirrhosis	40 (17.3%)	21 (19.1%)	11 (17.7%)	10 (20.8%)	
A	54 (23.4%)	20 (18.2%)	9 (14.5%)	11 (22.9%)	
B	63 (27.3%)	27 (24.5%)	13 (21.0%)	14 (39.2%)	
C	73 (31.6%)	41 (37.3%)	29 (46.8%)	12 (25.0%)	
Liver disease					0.125
Alcohol related liver disease	66 (28.6%)	35 (31.8%)	21 (33.9%)	14 (29.2%)	
Non-alcoholic steatohepatitis	20 (8.7%)	12 (10.9%)	8 (12.9%)	4 (8.3%)	
Viral hepatitis	56 (24.2%)	22 (20.0%)	9 (14.5%)	13 (27.1%)	
Biliary liver disease	22 (9.5%)	9 (8.2%)	8 (12.9%)	1 (2.1%)	
Other	58 (25.1%)	32 (29.1%)	16 (25.8%)	16 (33.3%)	
Acute liver failure	20 (8.7%)	13 (11.8%)	6 (9.7%)	7 (14.6%)	0.429
Carcinoma					0.346
HCC	96 (41.6%)	44 (40.0%)	25 (40.3%)	19 (39.6%)	
CCC	2 (0.9%)	2 (1.8%)	0	2 (4.2%)	
*Transplant procedure*					
Operation time (min)	270 (218–325)	235 (207–301)	236 (210–291)	233 (198–314)	0.995
Transfusion requirements					
EC	1 (0–3)	1 (0–3)	1 (0–3)	1 (0–3)	0.902
FFP	0 (0–0)	0 (0–0)	0 (0–2)	0 (0–0)	0.329
TC	0 (0–0)	0 (0–0)	0 (0–0)	0 (0–0)	0.337

Continuous variables are displayed as median and interquartile range

*EAD* earyl allograft dysfunction, *PNF* primary nonfunction, *BMI* body mass index, *DBD* donor after brain death, *DCD* donor after cardiac death, *RRT* renal replacement therapy, *HCC* hepatocellular carcinoma, *CCC* cholangiocell carcinoma

Baseline criteria of IRI patients are presented in Additional file 1: Table S1. As in the EAD group, there was a significant difference for DCD allografts (*p* < 0.001) and the preoperative MELD score (*p *= 0.039), while cold ischemic time did not yield significant differences.

Data concerning pre- and postoperative infections and immunosuppression are shown in Table 3. Preoperatively, there were no differences between EAD and non-EAD patients in terms of inflammatory laboratory parameters and controlled infections. During the 1st postoperative week, a total of 21 infections (19.1%) were diagnosed, with no significant difference between the EAD and non-EAD group [n = 10 (20.8%) and n = 11 (17.7%) respectively, *p* = 0.682]. No differences in immunosuppressive regimen and acute rejection episodes were found. Data for IRI patients are presented in Additional file 1: Table S2.

**Table 3:** Infections and immunosuppression in EAD versus non-EAD patients

	Whole cohort (n = 231)	Study cohort (n = 110)	Non-EAD (n = 62)	EAD (n = 48)	*p*-value
*Preoperative State*					
Recipient status					0.120
At home	158 (68.4%)	67 (60.9%)	34 (54.8%)	33 (68.8%)	
Admission general ward	42 (18.2%)	21 (19.1%)	16 (25.8%)	5 (10.4%)	
Admission on ICU	31 (13.4%)	22 (20%)	12 (19.4%)	10 (20.8%)	
Preoperative infection*	39 (16.%)	20 (18.2%)	13 (21.0%)	7 (14.6%)	0.389
Laboratory inflammatory parameters					
CRP (mg/l) (n = 109)	8.7 (3.0–22.0)	10 (4.1–24.0)	15 (3.8–28.5)	7.6 (4.2–16.8)	0.142
PCT (mcg/l) (n = 20)	1.1 (0.7–2.3)	1.2 (0.7–2.1)	1.4 (0.8–1.8)	1.0 (0.6–2.7)	0.656
WBC (G/l)	5.3 (3.7–7.0)	5.5 (3.9–7.4)	5.7 (4.5–7.5)	5.3 (3.3–7.4)	0.258
*Postoperative infections*					
Infection in first week	35 (15.2%)	21 (19.1%)	11 (17.7%)	10 (20.8%)	0.682
Donor transmitted Infection	5 (2.2%)	4 (3.6%)	2 (3.2%)	2 (4.2%)	
Respiratory tract infection	5 (2.2%)	2 (1.8%)	1 (1.6%)	1 (2.1%)	
Intraabdominal infection	8 (3.5%)	4 (3.6%)	2 (3.2%)	2 (4.2%)	
Urogenital infection	4 (1.7%)	3 (2.7%)	1 (1.6%)	2 (4.2%)	
Blood stream infection	2 (0.9%)	1 (0.9%)	1 (1.6%)	0	
Viral infection	3 (1.3%)	2 (1.8%)	1 (1.6%)	1 (2.1%)	
Other infection	12 (5.2%)	5 (4.5%)	3 (4.8%)	2 (4.2%)	
*Immunosuppression and rejection*					
Tacrolimus	222 (96.1%)	105 (95.5%)	61 (98.4%)	44 (91.7%)	0.093
Basiliximab	167 (72.3%)	81 (73.6%)	45 (72.6%)	36 (75.0%)	0.775
Acute rejection in first week	3 (1.3%)	2 (1.8%)	0	2 (4.2%)	0.188

Continous variables are displayed as median and interquartile range

*EAD* earyl allograft dysfunction, *CRP* C-reactive protein, *PCT* procalcitonin, *WBC* white blood cells

*Controlled infections under current antibiotic treatment

### PCT course during the first postoperative week (EAD and IRI)

**Fig. 2:**
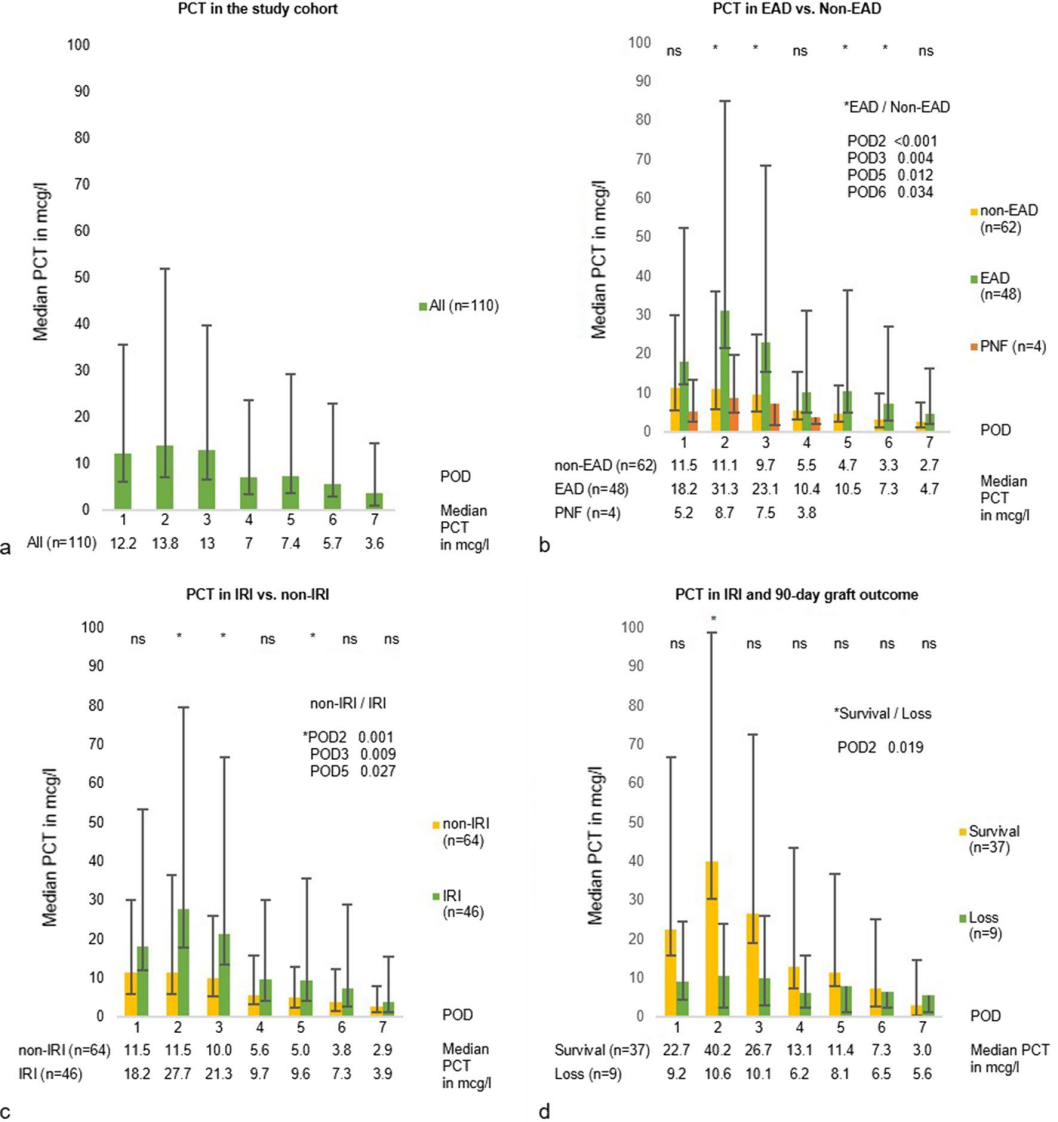
Procalcitonin during the first week after liver transplantation. Panel** A**. Whole study cohort. Panel **B**. EAD versus non-EAD patients. Panel **C**. IRI versus non-IRI patients. Panel **D**. IRI patients with graft survival versus IRI patients with graft loss. Bar charts represent median PCT level, IQR is represented by the vertical black line. P-values are indicated by ns (not significant) and * (significant)

Of the 110 OLT patients with a complete dataset of PCT on POD 1 + 2, PCT was available for 74, 54, 40, 32 and 30 patients on POD 3–7, respectively. A detailed presentation of missing PCT values is given in Additional file 1: Table S5. The overall PCT course in the study cohort of OLT patients is shown in Fig. 2a. PCT peaked during POD 1–3 with a steady decline afterwards. The highest PCT level of 13.8 (6.7–38.2) mcg/l was measured on POD 2.

Figure 2b and Additional file 1: Table S6 illustrate PCT courses in the non-EAD and EAD group. The PCT course in patients with PNF is illustrated separately in Fig. 2b. Peak PCT was observed within POD 1 + 2 in all groups. In the non-EAD group, highest PCT of 11.5 (6.0–18.6) mcg/l was noticed on POD 1. Overall, PCT values in the EAD group were significantly higher on POD 2, 3, 5 and 6 compared with those in the non-EAD group. On POD 2, the maximum difference in PCT was found with a median PCT peak of 31.3 (9.7–53.8) mcg/l in the EAD group compared to a median PCT of 11.1 (5.3–25.0) mcg/l in the non-EAD group (*p* < 0.001). PCT values for the PNF group were lower compared to the EAD and non-EAD group. On POD 2, a median maximum PCT of 8.7 (3.6–11.2) mcg/l was found in the PNF group. Due to the low case number, it was not possible to test this result for significance.

The PCT course for IRI and non-IRI patients is shown in Fig. 2c. PCT levels on POD 2, 3 and 5 in the IRI group were significantly higher compared to those in the non-IRI group, with a median maximum PCT of 27.7 (9.7–51.9) mcg/l versus 11.5 (5.5–25.2) mcg/l (*p* < 0.001) on POD 2.

Most patients fulfilling criteria for EAD (n = 48) according to Olthoff fulfilled the chosen criteria for IRI within the first 2 PODs (n = 46). As maximum PCT values were measured within POD 1 and 2, the predictive value of PCT for 90-day graft survival in the IRI patient cohort was investigated. The result is shown in Fig. 2d. IRI patients with 90-day graft survival had higher PCT values on POD 2 compared to IRI patients with 90-day graft loss [40.2 (9.8–58.6) mcg/l vs. 10.6 (8.2–13.4) mcg/l; *p* = 0.019].

### Short-term postoperative outcome, 90-day graft and patient survival

Data concerning postoperative outcome, 90-day graft and patient survival for EAD and non-EAD patients are presented in Table 4. Overall, 90-day graft survival was 88.2% and 90-day patient survival was 90.0% in the study cohort of OLT patients. EAD patients had a worse short-term outcome compared to patients without EAD: they displayed lower 90-day graft survival (79.2% vs. 95.2%; *p* = 0.01) and a trend towards lower 90-day patient survival (83.3% vs. 95.2%; *p* = 0.055). EAD patients had more arterial complications (12.5% vs. 1.6%; *p* = 0.042), had to undergo reoperation more often (54.2% vs. 29%; *p* = 0.008) and had a longer length of stay in the ICU [4 (2–9) days vs. 3 (2–5) days; *p* = 0.005].

**Table 4:** Short-term postoperative outcome, 90-day graft and patient survival of EAD and non-EAD patients

	Whole cohort (n = 231)	Study cohort (n = 110)	Non-EAD (n = 62)	EAD (n = 48)	*p*-value
Severity of IRI (peak ALT/AST)*	1358 (570–3191)	1506 (583–3881)	681 (334–1195)	4395 (3443–7605)	< 0.001
Overall major complication**	109 (47.2%)	65 (59.1%)	33 (53.2%)	32 (66.7%)	0.155
Reoperation	62 (26.8%)	44 (40%)	18 (29.0%)	26 (54.2%)	0.008
Postoperative RRT	96 (41.6%)	58 (52.7%)	29 (46.8%)	29 (60.4%)	0.155
Arterial complications	11 (4.8%)	7 (6.4%)	1 (1.6%)	6 (12.5%)	0.042
Hepatic artery thrombosis	5 (2.2%)	3 (2.7%)	0	3 (6.3%)	0.080
Biliary complications	37 (16%)	17 (15.5%)	9 (14.5%)	8 (16.7%)	0.757
ICU length of stay (days)	3 (2–5)	3 (2–5)	3 (2–5)	4 (2–9)	0.005
Hospital length of stay (days)	16 (12–23)	17 (13–22)	17 (13–22)	17 (13–24)	0.805
*90-day outcome*					
90-day graft survival	213 (92.2%)	97 (88.2%)	59 (95.2%)	38 (79.2%)	0.01
90-day patient survival	215 (93.1%)	99 (90.0%)	59 (95.2%)	40 (83.3%)	0.055

Continous variables are displayed as median and interquartile range

*EAD* earyl allograft dysfunction, *IRI* ischemia-reperfusion injury, *CRP* C-reactive protein, *PCT* procalcitonin, *ALT* alanine aminotransferase, *AST* aspartate aminotransferase, *RRT* renal replasement therapy

*Peak ALT/AST first 2 days

**Clavien-Dindo-stage ≥ 3B requiring intervention under general anesthesia, life-threatening complication requiring IC/ICU management, death of the patient

Data concerning postoperative outcome, 90-day graft and patient survival for IRI and non-IRI patients are given in Table 5. Likewise, IRI patients had a worse short-term outcome compared to non-IRI-patients: They displayed lower 90-day graft survival (80.4% vs. 93.8%; *p* = 0.033) with no difference in 90-day patient survival (*p* = 0.196). IRI patients had to undergo reoperation more often (54.3% vs. 29.7%; *p* = 0.009) and had a longer length of stay in the ICU [4 (2–8) days vs. 3 (2–5) days;* p* = 0.005]. As peak PCT was observed on POD 2 and a maximum difference in PCT level according to 90-day graft survival on this day was shown in IRI-patients (Fig. 2d), we chose to examine elevated transaminases (i.e. transaminases > 2000 IU/L; = the IRI group) as to whether PCT values on POD 2 might provide additional information on the outcome. A PCT threshold of 15 mcg/l was determined using the median of 13.8 mcg/l and the next higher value of 15.7 mcg/l in this cohort. Accordingly, the IRI group (n = 46) was divided into IRI patients with PCT > 15 mcg/l (n = 27) and IRI patients with PCT < 15 mcg/l (n = 19). Baseline characteristics as well as infections and immunosuppression of these two patient subgroups are shown in Additional file 1: Tables S3 and S4. IRI patients with PCT < 15 mcg/l on POD 2 had a worse outcome compared to non-IRI patients; that is, 90-day graft survival (57.9% vs. 93.8%; *p* = 0.001) and 90-day patient survival (68.4% vs. 93.8%; *p* = 0.008) was significantly decreased. They also had to undergo reoperation more often (68.4% vs. 29.7%; *p* = 0.002) and had a longer length of stay in the ICU [5 (3–14) days vs. 3 (2–5) days; *p* = 0.006]. In contrast, IRI patients with PCT > 15 mcg/l on POD 2 had no differences in short-term outcome parameters compared to non-IRI patients (*p* = 1.0).

**Table 5:** Short-term postoperative outcome, 90-day graft and patient survival of IRI and non-IRI patients

	Non-IRI (n = 64)	IRI (n = 46)	*p*-value	IRI and PCT > 15 mcg/l (n = 27)	*p*-value (vs. no IRI)	IRI and PCT < 15 mcg/l (n = 19)	*p*-value (vs. no IRI)
Severity of IRI (peak ALT/AST)*	681 (338–1250)	4563 (3108–7959)	< 0.001	4940 (3324–7607)	< 0.001	3721 (2960–9180)	< 0.001
Overall major complication**	34 (53.1%)	31 (67.4%)	0.133	18 (66.7%)	0.233	13 (68.4%)	0.237
Reoperation	19 (29.7%)	25 (54.3%)	0.009	12 (44.4%)	0.175	13 (68.4%)	0.002
Postoperative RRT	30 (46.9%)	28 (60.9%)	0.147	17 (63.0%)	0.161	11 (57.9%)	0.399
Arterial complications	2 (3.1%)	5 (10.9%)	0.127	2 (7.4%)	0.579	3 (15.8%)	0.076
Hepatic artery thrombosis	1 (1.6%)	2 (4.3%)	0.57	1 (3.7%)	0.508	1 (5.3%)	0.408
Biliary complications	9 (14.1%)	8 (17.4%)	0.634	3 (11.1%)	1.0	5 (26.3%)	0.293
ICU length of stay (days)	3 (2–5)	4 (2–8)	0.005	4 (2–6)	0.065	5 (3–14)	0.006
Hospital length of stay (days)	17 (13–22)	17 (13–24)	0.684	16 (13–18)	0.889	21 (11–35)	0.368
*90-day outcome*							
90-day graft survival	60 (93.8%)	37 (80.4%)	0.033	26 (96.3%)	1.0	11 (57.9%)	0.001
90-day patient survival	60 (93.8%)	39 (84.8%)	0.196	26 (96.3%)	1.0	13 (68.4%)	0.008

Continous variables are displayed as median and interquartile range

*EAD* earyl allograft dysfunction, *IRI* ischemia-reperfusion injury, *CRP* C-reactive protein, *PCT* procalcitonin, *ALT* alanine aminotransferase, *AST* aspartate aminotransferase, *RRT* renal replacement therapy

*Peak ALT/AST first 2 days

**Clavien-Dindo-stage ≥ 3B requiring intervention under general anesthesia, life-threatening complication requiring IC/ICU management, death of the patient

In all, 90-day graft loss occurred in eight IRI patients with PCT < 15 mcg/l. This was due to PNF in 4 cases, of which 3 underwent re-transplantation on POD 2 and 3. The other 4 graft losses were attributed to patient death because of multi-organ failure due to necrotizing pancreatitis (n = 2) or hemorrhagic shock (n = 2), of which one occurred after liver biopsy during workup for liver retransplantation. The 1 graft loss of an IRI patient with PCT > 15 mcg/l was due to patient death caused by thrombosis of the superior hepatic and inferior caval vein. The 4 graft losses in patients without IRI were due to patient death after a cerebral event (n = 2) or multiorgan failure, one after left hemihepatectomy due to malperfusion and one due to necrotizing pancreatitis.

### 12-month graft and patient survival

**Fig. 3:**
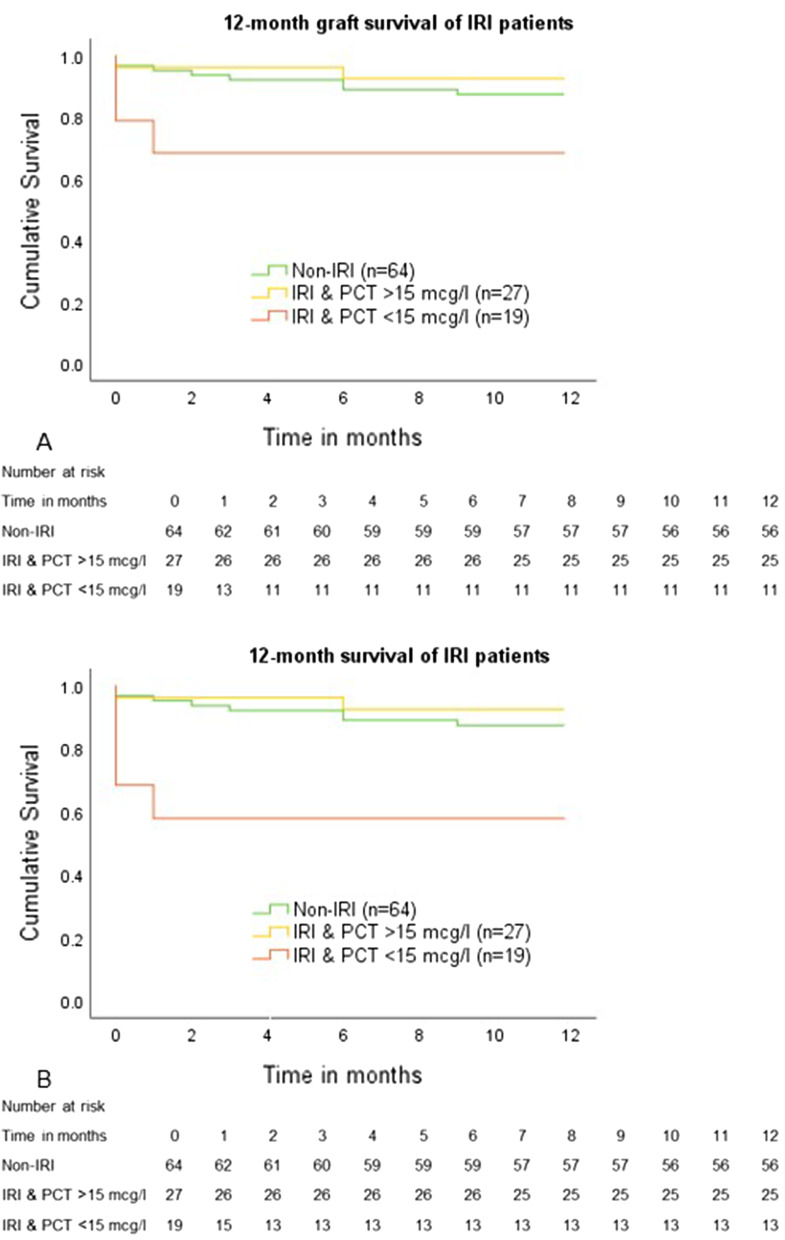
Kaplan Meier Analysis of graft and patient survival of non-IRI patients and IRI patients with PCT > 15 mcg/l versus IRI patients with PCT < 15 mcg/l. Panel **A**. 12-month graft survival of non-IRI patients was 56/64 (87.5%). 12-month graft survival of IRI patients with PCT > 15 mcg/l did not differ from that of non-IRI patients (25/27 (92.6%); p=0.483) and was lower in IRI patients with PCT < 15 mcg/l (11/19 (57.9%); p=0.002). Panel **B**. 12-month patient survival of non-IRI patients was 56/64 (87.5%). 12-month patient survival of IRI patients with PCT > 15 mcg/l did not differ from that of non-IRI patients (25/27 (92.6%); p = 0.483) and was lower in IRI patients with PCT < 15 mcg/l (13/19 (68.4%); p = 0.034)

Overall, 12-month graft and patient survival in the study cohort was 83.6% and 85.5%, respectively. There was no difference in graft and patient survival between the EAD and non-EAD group [graft survival: 55/62 (88.7%) vs. 37/48 (77.1%); *p* = 0.085/patient survival: 55/62 (88.7%) vs. 39/48 (81.3%); *p* = 0.240]. There was also no difference in graft and patient survival in IRI and non-IRI patients [graft survival: 56/64 (87.5%) vs. 36/46 (78.3%); *p* = 0.172/patient survival: 56/64 (87.5%) vs. 38/64 (82.6%); *p* = 0.437]. Patients however diagnosed with IRI and PCT < 15 mcg/l on POD 2 had a worse 12-month outcome compared to non-IRI patients and IRI patients with a PCT > 15 mcg/l on POD 2. Kaplan–Meier curves for 12-month graft and patient survival of these three groups are shown in Fig. 3a, b. Compared to the 56/64 (87.5%) of non-IRI patients with a functional graft at 12 months, IRI patients with PCT < 15 mcg/l on POD 2 had a functional graft in only 11/19 (57.9%) of cases (*p* = 0.002), while there was no difference in graft survival between non-IRI patients and IRI patients with PCT > 15 mcg/l on POD 2 [25/27 (92.6%); *p* = 0.483]. In addition, the 12-month survival rate of IRI patients with PCT < 15 mcg/l was significantly lower than that of non-IRI patients [13/19 (68.4%) vs. 56/64 (87.5%); *p* = 0.034], while there was no difference in 12-month patient survival between the IRI group with PCT > 15 mcg/l on POD 2 and the non-IRI group [25/27 (92.6%) vs. 56/64 (87.5%); *p* = 0.483].

## Discussion

While PCT synthesis in thyroidal C-cells during healthy states is well understood, knowledge about signalling pathways as well as PCT production and release in pathologic states is still accumulating [9]. Among various extra-thyroidal tissues and organs, the liver has been identified as the major source of PCT during bacterial infection [13, 15, 26]. While theoretically a decline in hepatic PCT production in extensive liver injury and dysfunction may be assumed, studies concerning PCT in advanced chronic liver disease and acute liver failure indicate a more complex relationship [27–30].

Earlier studies after OLT describe an early PCT peak with a steady decline in uncomplicated courses [17–21, 31–37]. Since PCT failed to predict infectious complications during the early postoperative phase [34, 35], higher PCT levels seem to be associated with a complicated postoperative course [17, 23, 32]. Our data for 110 adult OLT recipients confirm the previously described PCT peak on POD 1–3 with a following steady decline within the first postoperative week [17–21, 31–37] (Fig. 2a). They further showed, that PCT levels increase with post-transplant liver injury, but not with unfavorable outcomes (Fig. 2b, d).

Previous data concerning the association of PCT and EAD are contradictory: while Zant et al., reported PCT on POD 2 to correlate with aminotransferase elevations in paediatric liver transplantation [23], Eyraud et al. did not find an association of PCT with graft dysfunction (n = 12), in data from 67 liver transplanted patients [20]. We report continuously elevated PCT values in EAD patients during the first postoperative week with maximum PCT on POD 2 (*p* < 0.001) (Fig. 2b). Elevated aminotransferases in the setting of EAD are believed to be primarily associated with procedure-related IRI. Most EAD patients in our cohort fulfilled the Olthoff criteria by showing elevated transaminases on POD 1 or 2 (Tab. 1). We defined this elevation as a sign of IRI. Thus, for patients with EAD, we report continuously increased PCT in patients with signs of IRI during the first postoperative week, with maximum PCT on POD 2 (*p* < 0.001) (Fig. 2c). Interestingly, while IRI patients with PCT > 15 mcg/l had outcomes comparable to patients without IRI in terms of 90-day and 12-month graft and patient survival, the outcome in patients with IRI and PCT < 15 mcg/l was significantly worse (Tab. 5, Fig. 2d), suggesting a relevant role of PCT during the first postoperative days in patients with signs of severe post-transplant liver cell damage.

The PCT increase after OLT has several potential causes and pathways, which to date are not completely understood. It is known that endotoxins (EA) are released from the gut during the anhepatic phase of LT, a finding which has been associated with increased PCT levels [4, 17, 38]. Other cytokine-mediated inflammatory pathways that can be activated independent of infection by, e.g., major surgery, including IL-6, IL-8, TNF-α, IL-1β and TNF-α, are also known to increase PCT [15].

The serial pathophysiological process of ischemia and subsequent reperfusion of the donor organ during OLT, leading to ischemia-induced cell death and reperfusion-induced inflammation, is associated with EAD and PNF [8]. Briefly, the ischemia-induced mitochondrial dysfunction of hepatocytes leads to the production of reactive oxygen species (ROS), increasing tissue damage. This process is amplified on reperfusion, resulting in the release of damage-associated molecular patterns (DAMPs). DAMPs activate resident liver derived macrophages and thus induce infiltration of neutrophils, further enhancing the inflammatory cascade [7]. Both ROS and DAMPs stimulate PCT production.

Kupffer cells (KC) are an important part of the innate immune response and the largest fixed macrophage population in the body, accounting for 20–35% of total liver non-parenchymal cells [39]. In physiologic state, KC have been shown to maintain immune tolerance, while IRI may induce a phenotypic change of KC [40, 41]: The phenotypes of M1 macrophages have been shown to induce pro-inflammatory signalling, while M2 macrophages can counteract the pro-inflammatory process. During IRI, KC are polarized towards the proinflammatory M1 population [42], but the association with IRI so far has not been completely clarified [43]. Recently, KC have been identified as the hepatic source of PCT in an in vivo-model of acute liver failure, while hepatocytes were not found to produce PCT [44, 45]. Since the damage to hepatocytes is reflected in an increase in AST/ALT, PCT seems to reflect the stimulation of KC in the allograft. Moreover, KC may have a role in alleviating liver IRI by the production of IL-10 and terminating their own initial proinflammatory reaction [39]. PCT after OLT likely depends on a complex system of pro- and anti-inflammatory signalling. Multiple factors including recipient factors (e.g., pre-existing infection, age, comorbidities), donor factors (e.g., the procedure of organ donation (DCD/DBD), other donor specific characteristics) as well as procedure related factors (laparotomy, gut translocation, ischemia–reperfusion or transplant specific complications) impact PCT production.

Overall, PCT is significantly increased in patients with EAD or signs of IRI apart from the early postoperative peak noticed in all OLT patients. This finding suggests that the cascade of mechanisms associated with the development of IRI simultaneously induces a more intense stimulus presumably on KC, resulting in PCT increase [44].

Our data suggest that patients with IRI and PCT < 15 mcg/l had an impaired outcome in terms of graft and patient survival and four of the eight patients progressed to PNF. Meanwhile, IRI patients with PCT > 15 mcg/l had an outcome comparable to patients without IRI. Even though our results can only be hypothesis generating due to the study design and low case number, they point to PCT possibly being an indicator of KC function and therefore useful as a clinical monitoring parameter in the early post-transplant phase, which could help to guide decision making in cases of EAD and suspected PNF. An investigation concerning the association of PCT, PNF and outcome would thus be an interesting topic for a prospective study.

Our study has other limitations besides being a retrospective, single-centre study. As we decided to include patients with a complete PCT set on POD 1 and 2 only, there is a risk of both time-dependent and risk-dependent selection bias. PCT has increasingly been used in our ICU over the last few years, so PCT values have changed from use in complicated courses suspected of infection to more liberal use in uncomplicated courses as well. Baseline characteristics (Tables 2 and 3) concerning characteristics of the whole OLT cohort and the study cohort with PCT on POD 1 and 2 indicate, that more patients in the study group were treated in the ICU before transplantation and more patients with Child C cirrhosis were included in the study group. Furthermore, major complications according to Clavien-Dindo occurred more frequently in the study population compared to the whole study cohort. Overall, this indicates that PCT was measured more often in patients with a higher risk for complications. Baseline data do not suggest an association of PCT and early infections after OLT as another possible confounder. In addition the increased rate of reoperation and arterial complications in the EAD group might have influenced the different PCT level during the first postoperative week, but the aim of this study was not to investigate PCT as an independent risk factor, but to describe the association between PCT and EAD/IRI in a retrospective cohort of OLT. More data on these associations are required for deeper understanding.

## Conclusion

Generally, PCT is increased in the early postoperative phase after OLT. Patients with EAD and IRI have a significantly increased PCT maximum on POD 2, and impaired 90-day graft survival. PCT measurement may have potential as an additional outcome predictor in the early phase after OLT, as in our subanalysis of IRI patients, PCT values < 15 mcg/l were associated with impaired outcome.

## Supplementary Information

**Additional file 1: Table S1.** Baseline characteristics of IRI versus non-IRI patients. **Table S2.** Infections and immunosuppression of IRI versus non-IRI patients. **Table S3.** Baseline characteristics of IRI with PCT >15 mcg/l and IRI with PCT <15 mcg/l. **Table S4.** Infections and immunosuppression in IRI with PCT >15mcg/l and IRI with PCT <15 mcg/l. **Table S5.** Missing procalcitonin data on the basis of EAD and IRI. **Table S6.** Median PCT in EAD versus non-EAD during the first postoperative week. **Table S7.** Median PCT according to DCD and DBD in EAD and IRI. **Table S8. **Kidney function and renal replacement therapy in EAD versus non-EAD. **Table S9. **Kidney function and renal replacement therapy in IRI versus non-IRI. **Table S10.** Kidney function and renal replacement therapy in IRI with PCT >15 mcg/l and IRI with PCT <15 mcg/l.

## Data Availability

As data belong to the Institute of Intensive Care and Department of Surgery and Transplantation, University Hospital of Zurich, the datasets used and analyses during the current study are available from the corresponding author on reasonable request.

## Abbreviations

ALT: Alanine aminotransferase
AST: Aspartate aminotransferase
CRP: C-reactive protein
DAMPs: Damage associated molecular patterns
DBD: Donation after brain death
DCD: Donation after cardiac death
EA: Endotoxin
EAD: Early allograft dysfunction
GFR: Glomerular filtration rate
HOPE: Hypothermic oxygenated machine perfusion
ICU: Intensive care unit
INR: International normalized ratio
IRI: Ischemia–reperfusion injury
KC: Kupffer Cells
OLT: Orthotopic liver transplantation
PCT: Procalcitonin
POD: Postoperative day
PNF: Primary nonfunction
ROS: Reactive oxygen species
RRT: Renal replacement therapy
SOFA: Sequential organ failure assessment
