# Supplementary material for: Procalcitonin in early allograft dysfunction after orthotopic liver transplantation: a retrospective single centre study
Source: BMC Gastroenterol. 2022 Aug 31;22:404. doi: 10.1186/s12876-022-02486-5 (PMC9429388; doi:10.1186/s12876-022-02486-5)
Supplement: Supplementary file 1 — Additional file 1: Table S1. Baseline characteristics of IRI versus non-IRI patients. Table S2. Infections and immunosuppression of IRI versus non-IRI patients. Table S3. Baseline characteristics of IRI with PCT >15 mcg/l and IRI with PCT <15 mcg/l. Table S4. Infections and immunosuppression in IRI with PCT >15mcg/l and IRI with PCT <15 mcg/l. Table S5. Missing procalcitonin data on the basis of EAD and IRI. Table S6. Median PCT in EAD versus non-EAD during the first postoperative week. Table S7. Median PCT according to DCD and DBD in EAD and IRI. Table S8. Kidney function and renal replacement therapy in EAD versus non-EAD. Table S9. Kidney function and renal replacement therapy in IRI versus non-IRI. Table S10. Kidney function and renal replacement therapy in IRI with PCT >15 mcg/l and IRI with PCT <15 mcg/l. [file 12876_2022_2486_MOESM1_ESM.docx]

**Supplementary Material:**

**Procalcitonin in early allograft dysfunction after orthotopic liver transplantation:
A retrospective single centre study**

Katja Frick^1^, MD, Elisabeth A. Beller^1^, MD, Marit Kalisvaart^2^, MD PhD
Phillipp Dutkowski^2^, MD, Reto A. Schüpbach^1^, MD, Stephanie Klinzing^1^, MD

^1^ Institute of Intensive Care, University Hospital of Zurich, Switzerland
^2^ Department of Surgery and Transplantation, University Hospital of Zurich, Switzerland

**Supplemantary tables**

Table S1. Baseline characteristics of IRI versus non-IRI patients………………………….3

Table S2. Infections and immunosuppression of IRI versus non-IRI patients…………….4

Table S3. Baseline characteristics of IRI with PCT >15 mcg/l and IRI with
PCT <15 mcg/l…………………………………………………………………….....5

Table S4. Infections and immunosuppression in IRI with PCT >15mcg/l and IRI with PCT <15 mcg/l ………………………………………............………....…………..6

Table S5. Missing procalcitonin data on the basis of EAD and IRI……...………………...7

Table S6. Median PCT in EAD versus non-EAD during the first postoperative week………………………………………………….............................................8

Table S7. Median PCT according to DCD and DBD in EAD and IRI………...……...........9

Table S8. Kidney function and renal replacement therapy in EAD versus non-EAD…...10

Table S9. Kidney function and renal replacement therapy in IRI versus non-IRI……….11

Table S10. Kidney function and renal replacement therapy in IRI with PCT >15 mcg/l
and IRI with PCT <15 mcg/l…………………..…………………………………..12

**Table S1.** Baseline characteristics of IRI versus non-IRI patients

|  | **Whole cohort (n=231)** | **Study group (n=110)** | **Non-IRI (n=64)** | **IRI (n=46)** | **p-value** |
| --- | --- | --- | --- | --- | --- |
| **Donor & Graft** |  |  |  |  |  |
| Age (years) | 58 (47-71) | 60 (48-73) | 60 (48-72) | 61 (50-73) | 0.662 |
| Female gender | 98 (42.4%) | 47 (43%) | 31 (48.4%) | 16 (34.8%) | 0.153 |
| BMI (n=109) | 25.5 (23.0-28.0) | 26 (23-28) | 25 (22-28) | 26 (24-29) | 0.341 |
| Split liver transplantation | 7 (3.0%) | 3 (3%) | 3 (4.7%) | 0 | 0.263 |
| DCD | 68 (29.4%) | 31 (28%) | 8 (12.5%) | 22 (47.8%) | <0.001 |
| Cold ischemia time (h) (n=106) | 7.0 (5.8-8.4) | 7.1 (5.8-8.6) | 6.6 (5.4-8.5) | 7.4 (6.5-9.1) | 0.052 |
| Warm ischemia time (min) | 34.0 (30.0-38.0) | 34.5 (29.8-38.3) | 34.5 (31.5-44.0) | 33.5 (29.0-38.25) | 0.504 |
| Machine perfusion (HOPE) | 116 (50.2%) | 60 (55%) | 30 (46.9%) | 30 (65.2%) | 0.057 |
| **Recipient** |  |  |  |  |  |
| Age (years) | 57 (48-63) | 57 (48-64) | 57 (46-63) | 56 (50-64) | 0.879 |
| Female gender | 76 (32.9%) | 36 (33%) | 19 (29.7%) | 17 (37.0%) | 0.423 |
| Body mass index | 27.0 (22.7-30.1) | 27.4 (23.5-30.8) | 27.3 (23.0-30.7) | 27.9 (24.0-31.5) | 0.839 |
| Charlson-Comorbidity-Index | 5 (3-6) | 5 (3-5) | 5 (3-5) | 5 (3-5) | 0.702 |
| Serum-Creatinine (µmol/l) (n=81) | 88.0 (67.5-133.0) | 93.0 (67.5-145.0) | 90.0 (70.8-155.3) | 94.0 (66.0-124.0) | 0.434 |
| Preoperative RRT | 53 (22.9%) | 28 (25.5%) | 18 (28.1%) | 10 (21.7%) | 0.510 |
| Laboratory MELD-score (n=109)) | 19 (11-31) | 20 (12-33) | 23 (15-34) | 17 (9-32) | 0.039 |
| Liver cirrhosis (Child-Pugh-Score) | |  |  |  | 0.114 |
| No cirrhosis | 40 (17.3%) | 21 (19%) | 11 (17.2%) | 10 (21.7%) |  |
| A | 54 (23.4%) | 20 (18%) | 9 (14.1%) | 11 (23.9%) |  |
| B | 63 (27.3%) | 27 (25%) | 14 (21.9%) | 13 (28.3%) |  |
| C | 73 (31.6%) | 41 (38%) | 30 (46.9%) | 11 (23.9%) |  |
| Liver disease |  |  |  |  | 0.037 |
| Alcohol related liver disease | 66 (28.6%) | 35 (32%) | 21 (32.8%) | 14 (30.4%) |  |
| Non-alcoholic steatohepatitis | 20 (8.7%) | 12 (11%) | 10 (15.6%) | 2 (4.3%) |  |
| Viral hepatitis | 56 (24.2%) | 22 (20%) | 9 (14.1%) | 13 (28.3%) |  |
| Biliary liver disease | 22 (9.5%) | 9 (8%) | 8 (12.5%) | 1 (2.2%) |  |
| Other | 58 (25.1%) | 32 (29%) | 16 (25%) | 16 (34.8%) |  |
| Acute liver failure | 20 (8.7%) | 13 (12%) | 6 (9.4%) | 7 (15.2%) | 0.349 |
| Carcinoma |  |  |  |  | 0.335 |
| HCC | 96 (41.6%) | 44 (40%) | 26 (40.6%) | 18 (39.1%) |  |
| CCC | 2 (0.9%) | 2 (2%) | 0 | 2 (4.3%) |  |
| **Transplant procedure** |  |  |  |  |  |
| Operation time (min) | 270 (218-325) | 3.9 (3.5-5.0) | 241 (211-295) | 230 (197-308) | 0.544 |
| Transfusion requirements |  |  |  |  |  |
| EC | 1 (0-3) | 1 (0-3) | 1 (0-4) | 1 (0-2) | 0.809 |
| FFP | 0 (0-0) | 0 (0-3) | 0 (0-2) | 0 (0-0) | 0.193 |
| TC | 0 (0-0) | 0 (0-3) | 0 (0-0) | 0 (0-0) | 0.442 |
| IRI, ischemia-reperfusion-injury; PNF, primary nonfunction; HOPE, hypothermic oxygenated liver perfusion; BMI, body mass index; DBD, donor after brain death; DCD, donor after cardiac death; RRT, renal replacement therapy; HCC, hepatocellular carcinoma; CCC, cholangiocell carcinoma. Continous variables are displayed as median and interquartile range. | | | | | |

**Table S2.** Infections and immunosuppression of IRI versus non-IRI patients

|  | **Whole cohort (n=231)** | **Study group (n=110)** | **no IRI (n=64)** | **IRI (n=46)** | **p-value** |
| --- | --- | --- | --- | --- | --- |
| **Preoperative State** |  |  |  |  |  |
| Recipient status |  |  |  |  | 0.151 |
| At home | 158 (68.4%) | 67 (60.9%) | 35 (54.7%) | 32 (69.6%) |  |
| Admission general ward | 42 (18.2%) | 21 (19.1%) | 16 (25%) | 5 (10.9%) |  |
| Admission on ICU | 31 (13.4%) | 22 (20%) | 13 (20.3%) | 9 (19.6%) |  |
| Preoperative infection* | 39 (16.%) | 20 (18.2%) | 14 (21.9%) | 6 (13%) | 0.236 |
| Laboratory inflammatory parameters | |  |  |  |  |
| CRP (mg/l) (n=109) | 8.7 (3.0-22.0) | 10 (4.1-24) | 15 (3.9-29.0) | 7.6 (4.2-16.3) | 0.147 |
| PCT (mcg/l) (n=20) | 1.1 (0.7-2.3) | 1.2 (0.7-2.1) | 1.4 (0.8-1.7) | 0.9 (0.6-2.3) | 0.624 |
| WBC (G/l) | 5.3 (3.7-7.0) | 5.5 (3.9-7.4) | 5.7 (4.5-7.6) | 5.3 (3.3-7.1) | 0.216 |
| **Postoperative infections** |  |  |  |  |  |
| Infection first week | 35 (15.2%) | 21 (19.1%) | 11 (17.2%) | 10 (21.7%) | 0.549 |
| Donor transmitted Infection | 5 (2.2%) | 4 (3.6%) | 2 (3.1%) | 2 (4.3%) |  |
| Respiratory tract infection | 5 (2.2%) | 2 (1.8%) | 1 (1.6%) | 1 (2.2%) |  |
| Intraabdominal infection | 8 (3.5%) | 4 (3.6%) | 2 (3.1%) | 2 (4.3%) |  |
| Urogenital infection | 4 (1.7%) | 3 (2.7%) | 1 (1.6%) | 2 (4.3%) |  |
| Blood stream infection | 2 (0.9%) | 1 (0.9%) | 1 (1.6%) | 0 |  |
| Viral infection | 3 (1.3%) | 2 (1.8%) | 1 (1.6%) | 1 (2.2%) |  |
| Other infection | 12 (5.2%) | 5 (4.5%) | 3 (4.7%) | 2 (4.3%) |  |
| **Immunosuppression & Rejection** | |  |  |  | |
| Tacrolimus | 222 (96.1%) | 105 (95.5%) | 63 (98.4%) | 42 (91.3%) | 0.159 |
| Basiliximab | 167 (72.3%) | 81 (73.6%) | 47 (73.4%) | 34 (73.9%) | 0.955 |
| Acute rejection in first week | 3 (1.3%) | 2 (1.8%) | 0 | 2 (4.3%) | 0.173 |
| EAD, earyl allograft dysfunction; CRP, C-reactive protein; PCT, procalcitonin; WBC White blood cells. Continous variables are displayed as median and interquartile range. | | | | | |
| ** Controlled infections under current antibiotic treatment* | | | | | |

**Table S3.** Baseline characteristics of IRI with PCT >15 mcg/l and IRI with PCT <15 mcg/l

|  | **IRI & PCT >15 mcg/l (n=27)** | **IRI & PCT <15 mcg/l (n=19)** | **p-value** |
| --- | --- | --- | --- |
| **Donor & Graft** |  |  |  |
| Age (years) | 60 (39-64) | 73 (56-80) | 0.010 |
| Female gender | 7 (26%) | 9 (47%) | 0.209 |
| BMI (n=109) | 26 (24-29) | 26 (25-29) | 0.686 |
| Split liver transplantation | 0 | 0 | / |
| DCD | 13 (48%) | 9 (47%) | 1.000 |
| Cold ischemia time (h) (n=45) | 7.3 (6.8-9.4) | 7.4 (6.6-8.4) | 0.694 |
| Warm ischemia time (min) (n=22) | 32.0 (29.5-38.0) | 36.0 (28.5-39.0) | 0.713 |
| Machine perfusion (HOPE) | 18 (67%) | 12 (63%) | 1.000 |
| **Recipient** |  |  |  |
| Age (years) | 54 (49-64) | 59 (52-63) | 0.517 |
| Female gender | 11 (41%) | 6 (32%) | 0.555 |
| Body mass index | 27.5 (23.7-29.5) | 28.4 (24.1-33.1) | 0.462 |
| Charlson-Comorbidity-Index | 5 (3-5) | 3 (3-6) | 0.918 |
| Serum-Creatinine (µmol/l) (n=35) | 102.0 (70.8-121.3) | 80.0 (55.0-137.0) | 0.371 |
| Preoperative RRT (n=10) | 6 (22.2%) | 4 (21.1%) | 1.000 |
| Laboratory MELD-score (n=45) | 17 (11-33) | 17 (8-31) | 0.581 |
| Liver cirrhosis (Child-Pugh-Score) | |  | 0.161 |
| No cirrhosis | 4 (15%) | 6 (33%) |  |
| A | 5 (19%) | 6 (33%) |  |
| B | 9 (33%) | 4 (22%) |  |
| C | 9 (33%) | 2 (11%) |  |
| Liver disease |  |  | 0.563 |
| Alcohol related liver disease | 7 (26%) | 7 (37%) |  |
| Non-alcoholic steatohepatitis | 2 (7%) | 0 |  |
| Viral hepatitis | 9 (33%) | 4 (21%) |  |
| Biliary liver disease | 1 (4%) | 0 |  |
| Other | 8 (30%) | 8 (42%) |  |
| Acute liver failure | 3 (11%) | 4 (21%) | 0.424 |
| Carcinoma |  |  | 0.882 |
| HCC | 10 (37%) | 8 (42%) |  |
| CCC | 1 (4%) | 1 (5%) |  |
| **Transplant procedure** |  |  |  |
| Operation time (min) | 220 (197-287) | 261 (196-328) | 0.337 |
| Transfusion requirements |  |  |  |
| EC | 1 (1-3) | 0 (0-2) | 0.100 |
| FFP | 0 (0-0) | 0 (0-0) | 0.787 |
| TC | 0 (0-0) | 0 (0-0) | 0.748 |
| IRI, ischemia-reperfusion-injury; PNF, primary nonfunction; HOPE, hypothermic oxygenated liver perfusion; BMI, body mass index; DBD, donor after brain death; DCD, donor after cardiac death; RRT, renal replacement therapy; HCC, hepatocellular carcinoma; CCC, cholangiocell carcinoma. Continous variables are displayed as median and interquartile range. | | | |

**Table S4.** Infections and immunosuppression in IRI with PCT >15mcg/l and IRI with PCT <15 mcg/l

|  | **IRI & PCT >15 mcg/l (n=27)** | **IRI & PCT <15 mcg/l (n=19)** | **p-value** |
| --- | --- | --- | --- |
| **Preoperative State** |  |  |  |
| Recipient status |  |  | 1.000 |
| At home | 19 (70.4%) | 13 (68.4%) |  |
| Admission general ward | 3 (11.1%) | 2 (10.5%) |  |
| Admission on ICU | 5 (18.5%) | 4 (21.1%) |  |
| Preoperative infection* | 4 (14.8%) | 2 (10.5%) | 1.000 |
| Laboratory inflammatory parameters | |  |  |
| CRP (mg/l) (n=109) | 10.0 (4.9-21.0) | 6.3 (3.0-11.5) |  |
| PCT (mcg/l) (n=20) | 2.3 (1.4-9.7) | 0.8 (0.6-0.9) |  |
| WBC (G/l) | 5.3 (3.3-6.6) | 5.2 (3.7-8.0) |  |
| **Postoperative infections** |  |  |  |
| Infection first week | 5 (18.5%) | 5 (26.3%) | 0.719 |
| Donor transmitted Infection | 1 (3.7%) | 1 (5.3%) |  |
| Respiratory tract infection | 1 (3.7%) | 0 |  |
| Intraabdominal infection | 0 | 2 (10.5%) |  |
| Urogenital infection | 1 (3.7%) | 1 (5.3%) |  |
| Blood stream infection | 0 | 0 |  |
| Viral infection | 0 | 1 (5.3%) |  |
| Other infection | 2 (7.4%) | 0 |  |
| **Immunosuppression & Rejection** | |  |  |
| Tacrolimus | 27 (100%) | 15 (78.9%) | 0.024 |
| Basiliximab | 22 (81.5%) | 12 (63.2%) | 0.190 |
| Acute rejection in first week | 1 (3.7%) | 1 (5.3%) | 1.000 |
| EAD, earyl allograft dysfunction; CRP, C-reactive protein; PCT, procalcitonin; WBC White blood cells. Continous variables are displayed as median and interquartile range. | | | |
| ** Controlled infections under current antibiotic treatment* | | | |

**Table S5.** Missing procalcitonin data on the basis of EAD and IRI

|  | | |  |  |  |  |  |  |  |  |  |  |  |  |
| --- | --- | --- | --- | --- | --- | --- | --- | --- | --- | --- | --- | --- | --- | --- |
|  | **Whole cohort (n=231)** | | **Study cohort (n=110)** | | **EAD (n=62)** | | **non EAD (n=48)** | | **p-value** | **IRI (n=64)** | | **non IRI (n=46)** | | **p-value** |
|  | **PCT existent** | **PCT missing** | **PCT existent** | **PCT missing** | **PCT existent** | **PCT missing** | **PCT existent** | **PCT missing** |  | **PCT existent** | **PCT missing** | **PCT existent** | **PCT missing** |  |
| POD1 | 137 (59.3%) | 94 (40.7%) | 110 (100%) | 0 | 62 (100%) | 0 | 48 (100%) | 0 |  | 64 (100%) | 0 | 46 (100%) | 0 |  |
| POD2 | 124 (53.7%) | 107 (46.3%) | 110 (100%) | 0 | 62 (100%) | 0 | 48 (100%) | 0 |  | 64 (100%) | 0 | 46 (100%) | 0 |  |
| POD3 | 86 (37.2%) | 145 (62.8%) | 74 (64.3%) | 36 (32.7%) | 34 (70.8%) | 14 (29.2%) | 40 (64.5%) | 22 (35.5%) | 0.484 | 33 (71.7) | 13 (28.3%) | 41 (64.1%) | 23 (35.9%) | 0.397 |
| POD4 | 67 (29%) | 164 (71%) | 54 (49.1%) | 56 (50.9%) | 28 (58.3%) | 20 (41.7%) | 26 (41.9%) | 36 (58.1%) | 0.088 | 27 (58.7%) | 19 (41.3%) | 27 (42.2%) | 37 (57.8%) | 0.088 |
| POD5 | 48 (20.8%) | 183 (79.2%) | 40 (36.4%) | 70 (63.6%) | 24 (50%) | 24 (50%) | 16 (25.8%) | 46 (74.2%) | 0.009 | 23 (50%) | 23 (50%) | 17 (26.6%) | 47 (73.4%) | 0.012 |
| POD6 | 42 (18.2%) | 189 (81.8%) | 32 (29.1%) | 78 (70.9%) | 18 (37.5%) | 30 (62.5%) | 14 (22.7%) | 48 (77.4%) | 0.088 | 17 (37%) | 29 (63%) | 15 (23.4%) | 49 (76.6%) | 0.124 |
| POD7 | 37 (16%) | 194 (84%) | 30 (27.3%) | 80 (72.7%) | 16 (33.3%) | 32 (66.7%) | 14 (22.6%) | 48 (77.4%) | 0.209 | 15 (32.6%) | 31 (67.4%) | 15 (23.4%) | 49 (76.6%) | 0.287 |
| POD, postoperative day; PCT, procalcitonin; EAD, early allograft dysfunction; IRI, ischemia-reperfusion injury. | | | | | | | | | | | | | | |

**Table S6.** Median PCT in EAD versus non-EAD during the first postoperative week

| **POD** | **1** | **2** | **3** | **4** | **5** | **6** | **7** |
| --- | --- | --- | --- | --- | --- | --- | --- |
| **n** | **110** | **110** | **74** | **54** | **40** | **32** | **30** |
| **Non-EAD (n=62)** | **11.5** | **11.1** | **9.7** | **5.5** | **4.7** | **3.3** | **2.7** |
| IQR | 6.0-18.6 | 5.3-25.0 | 4.4-15.5 | 2.1-9.9 | 2.0-7.2 | 39601.0 | 1.4-5.0 |
| **EAD (n=48)** | **18.2** | **31.3** | **23.1** | **10.4** | **10.5** | **7.3** | **4.7** |
| IQR 25 | 5.9-34.4 | 9.7-53.8 | 7.7-45.5 | 5.5-20.8 | 5.4-25.9 | 4.4-19.9 | 2.7-11.6 |
| **p-value** | **0.073** | **<0.001** | **0.004** | **0.056** | **0.012** | **0.034** | **0.070** |
| POD, postoperative day; EAD, early allograft dysfunction; IQR, interquartile range. Continuous variables are displayed as median and interquartile range. | | | | | | | |

**Table S7.** Median PCT according to DCD and DBD in EAD and IRI

| **PCT in DCD versus DBD of the study cohort (n=110)** | | | | | | | |
| --- | --- | --- | --- | --- | --- | --- | --- |
| POD | 1 | 2 | 3 | 4 | 5 | 6 | 7 |
| n | 110 | 110 | 74 | 54 | 40 | 32 | 30 |
| DCD (n=30) | 18.7 | 26.4 | 16.8 | 7.1 | 7.8 | 6.5 | 3.1 |
| IQR | 6.2-35.2 | 10.4-58.2 | 9.2-45.4 | 5.4-22.6 | 4.4-29.7 | 2.9-21.0 | 1.4-5.2 |
| DBD (n=80) | 11.7 | 11.8 | 9.2 | 6.2 | 7.3 | 5.4 | 3.6 |
| IQR | 6.0-20.0 | 6.2-31.6 | 5.4-24.8 | 2.6-15.2 | 3.5-21.8 | 2.8-17.2 | 2.4-11.4 |
| p-value | 0.137 | 0.012 | 9.2 | 0.436 | 0.561 | 0.782 | 0.321 |
| **PCT in DCD versus DBD in Non-EAD patients (n=62)** | | | | | | | |
| POD | 1 | 2 | 3 | 4 | 5 | 6 | 7 |
| n | 62 | 62 | 40 | 26 | 16 | 14 | 14 |
| DCD (n=8) | 20.7 | 25.7 | 14.2 | 8.7 | 5.7 | 4.1 | 3.3 |
| IQR | 8.7-40.4 | 13.8-44.4 | 10.6-24.0 | 5.8-22.1 | 4.2-/ | 2.5-/ | 1.6-/ |
| DBD (n=54) | 10.5 | 10.6 | 9.0 | 4.9 | 4.7 | 3.3 | 2.7 |
| IQR | 4.6-16.7 | 4.9-21.4 | 3.3-14.5 | 1.7-9.6 | 1.4-7.5 | 1.3-8.1 | 1.0-8.1 |
| p-value | 0.042 | 0.031 | 0.109 | 0.197 | 0.700 | 1.000 | 1.000 |
| **PCT in DCD versus DBD in EAD patients (n=48)** | | | | | | | |
| POD | 1 | 2 | 3 | 4 | 5 | 6 | 7 |
| n | 48 | 48 | 34 | 28 | 24 | 18 | 16 |
| DCD (n=22) | 17.2 | 30.9 | 21.3 | 7.1 | 12.4 | 7.3 | 3.1 |
| IQR | 4.8-35.2 | 9.4-60.7 | 8.5-53.1 | 4.6-26.5 | 4.4-50.7 | 3.4-42.2 | 1.1-5.3 |
| DBD (n=26) | 18.2 | 31.3 | 24.8 | 11.7 | 10.5 | 42.2 | 8.1 |
| IQR | 6.5-37.7 | 9.8-48.2 | 7.6-39.5 | 5.8-20.8 | 5.8-25.4 | 4.6-17.9 | 2.8-13.4 |
| p-value | 0.756 | 0.812 | 0.675 | 0.735 | 0.820 | 1.000 | 0.133 |
| **PCT in DCD versus DBD in Non-IRI patients (n=64)** | | | | | | | |
| POD | 1 | 2 | 3 | 4 | 5 | 6 | 7 |
| n | 64 | 64 | 41 | 27 | 17 | 15 | 15 |
| DCD (n=8) | 20.7 | 25.7 | 14.2 | 8.7 | 5.7 | 4.1 | 3.3 |
| IQR | 8.7-40.4 | 13.8-44.4 | 10.6-24.0 | 5.8-22.1 | 4.2-/ | 2.5-/ | 1.6-/ |
| DBD (n=56) | 10.5 | 10.8 | 9.0 | 5.0 | 5.0 | 3.8 | 2.9 |
| IQR | 4.6-17.8 | 5.1-24.3 | 3.4-15.8 | 1.8-9.7 | 1.5-8.8 | 1.7-11.0 | 1.4-7.9 |
| p-value | 0.044 | 0.049 | 0.138 | 0.272 | 0.824 | 0.933 | 0.933 |
| **PCT in DCD versus DBD in IRI patients (n=46)** | | | | | | | |
| POD | 1 | 2 | 3 | 4 | 5 | 6 | 7 |
| n | 46 | 46 | 33 | 27 | 23 | 17 | 15 |
| DCD (n=22) | 17.2 | 30.9 | 21.3 | 7.1 | 12.4 | 7.3 | 3.1 |
| IQR | 4.8-35.2 | 9.4-60.7 | 8.5-53.1 | 4.6-26.5 | 4.4-50.7 | 3.4-42.2 | 1.1-5.3 |
| DBD (n=24) | 18.2 | 27.6 | 22.7 | 11.4 | 9.6 | 5.7 | 5.4 |
| IQR | 6.9-41.6 | 9.7-48.1 | 7.6-42.5 | 5-20.7 | 5.1-25.6 | 4.5-18.0 | 2.7-14.0 |
| p-value | 0.692 | 0.636 | 0.624 | 0.820 | 0.759 | 1.000 | 0.177 |
| PCT, procalcitonin; DCD, donation after cardiac death; DBD, donation after brain death; EAD, early allograft dysfunction; IRI, ischemia-reperfusion injury; POD, postoperative day; IQR, interquartile range. | | | | | | | |
|  |  |  |  |  |  |  |  |

**Table S8.** Kidney function and renal replacement therapy in EAD versus non-EAD

|  | **Whole cohort (n=231)** | **Study cohort (n=110)** | **Non-EAD (n=62)** | **EAD (n=48)** | **p-value** |
| --- | --- | --- | --- | --- | --- |
| Serum-Creatinine (µmol/l) (n=81) | 88 (68-133) | 93 (68-145) | 91 (71-156) | 94 (66-122) | 0.384 |
| Preoperative RRT | 53 (22.9%) | 28 (25.5%) | 17 (27.4%) | 11 (22.9%) | 0.591 |
| Postoperative RRT | 96 (41.6%) | 58 (52.7%) | 29 (46.8%) | 29 (60.4%) | 0.155 |
| *Postoperative AKI requiring RRT* | 51 (22.1%) | 32 (29.1%) | 13 (21%) | 19 (39.6%) | 0.037 |
| RRT, renal replacement therapy; EAD, early allograft dysfunction. AKI, acute kidney injury. Continous variables are displayed as median and interquartile range. | | | | | |

**Table S9.** Kidney function and renal replacement therapy in IRI versus non-IRI

|  | **Whole cohort (n=231)** | **Study group (n=110)** | **Non-IRI (n=64)** | **IRI (n=46)** | **p-value** |
| --- | --- | --- | --- | --- | --- |
| Serum-Creatinine (µmol/l) (n=81) | 88.0 (67.5-133.0) | 93.0 (67.5-145.0) | 90.0 (70.8-155.3) | 94.0 (66.0-124.0) | 0.434 |
| Preoperative RRT | 53 (22.9%) | 28 (25.5%) | 18 (28.1%) | 10 (21.7%) | 0.510 |
| Postoperative RRT | 96 (41.6%) | 58 (52.7%) | 30 (46.9%) | 28 (60.9%) | 0.147 |
| *Postoperative AKI requiring RRT* | 51 (22.1%) | 32 (29.1%) | 13 (20.3%) | 19 (42.3%) | 0.020 |
| RRT, renal replacement therapy; EAD, early allograft dysfunction. AKI, acute kidney injury. Continous variables are displayed as median and interquartile range. | | | | | |

**Table S10.** Kidney function and renal replacement therapy in IRI with PCT >15 mcg/l and IRI with PCT <15 mcg/l

|  | **IRI & PCT >15 mcg/l (n=27)** | **IRI & PCT <15 mcg/l (n=19)** | **p-value** |
| --- | --- | --- | --- |
| Serum-Creatinine (µmol/l) (n=35) | 102.0 (70.8-121.3) | 80.0 (55.0-137.0) | 0.371 |
| Preoperative RRT | 6 (22.2%) | 4 (21.1%) | 1.000 |
| Postoperative RRT | 17 (63.0%) | 11 (57.9%) | 0.729 |
| *Postoperative AKI requiring RRT* | 11 (40.7%) | 8 (42.1%) | 0.926 |
| RRT, renal replacement therapy; EAD, early allograft dysfunction. AKI, acute kidney injury. Continous variables are displayed as median and interquartile range. | | | |
